# HPV52 predominates in cervical infections and precancerous lesions in Chongqing, China: a 6-year study linking genotypes to vaginal microecology

**DOI:** 10.3389/fpubh.2026.1879232

**Published:** 2026-08-14

**Authors:** Xue Li, Xueqin Fan, Tingjia Chai, Liyi Hu, XuanLin Lu, Sai Ren

**Affiliations:** 1 Department of Obstetrics and Gynecology, Chongqing Health Center for Women and Children, Chongqing, China; 2 Department of Obstetrics and Gynecology, Women and Children's Hospital of Chongqing Medical University, Chongqing, China; 3 Chongqing Municipal Health Commission Key Laboratory of Perinatal Medicine, Chongqing, China; 4 Department of Laboratory Medicine, Chongqing Western Hospital, Chongqing, China; 5 Endocrinology Laboratory, The Second Affiliated Hospital of Chongqing Medical University, Chongqing, China; 6 Department of Laboratory Medicine, People's Hospital of Chongqing Liang Jiang New Area, Chongqing, China

**Keywords:** cervical cancer, cervical lesions, HPV, prevalence, vaginal microecology

## Abstract

**Background:**

Cervical cancer continues to pose a considerable public health burden in Southwest China, yet region-specific data on HPV genotypes, age-related patterns, and their associations with vaginal microecology are limited.

**Methods:**

From 2019 to 2024, 24,050 women aged 18–80 years were recruited at the People’s Hospital of Liangjiang New Area, Chongqing. All participants underwent HPV genotyping for 23 subtypes using PCR reverse dot blot hybridization, ThinPrep cytology test (TCT), and vaginal microecological evaluation. This integrated detection strategy allowed us to further analyze the overall HPV positive rate, age-specific genotype distribution, the correlation between HPV infection and cervical lesions, and the interactive relationship between HPV infection and vaginal microecology.

**Results:**

The overall detection rate of HPV was 21.7% (5,211/24,050), increasing from 7.5% (2019) to 30.1% (2022) and remaining stable at ~25% throughout 2023–2024. HPV52 was the predominant genotype in single (19.9%, 690/4375) and multiple (11.2%, 493/4418) infections, with HPV16 and HPV53 ranking closely behind. HPV prevalence peaked at ≤20 years (40.3%) and >60 years (39.2%). HPV16 positivity increased with lesions severity (LSIL: 18.3%, HSIL: 36.1%, SCC: 60.9%). In contrast, HPV52 was mainly associated with precancerous lesions (LSIL: 26.6%, ASCUS: 26.0%). Additionally, multiple infections were associated with a higher prevalence of abnormal cytology. Women diagnosed with vulvovaginal candidiasis (VVC), bacterial vaginosis (BV) and trichomoniasis vaginitis (TV) had significantly higher HPV52/16 positivity than controls. High-risk HPV (HR-HPV) positivity was correlated with poorer vaginal cleanliness (*p* < 0.001).

**Conclusion:**

HPV52 predominates in infections and precancerous lesions in Chongqing. Vaginal microecological disturbance was strongly associated with HR-HPV infection. HPV16 was enriched in severe dysbiosis (Grade III and IV), whereas HPV52 showed broader associations with BV, TV and VVC. These results suggest that the 9-valent vaccine, which covers HPV52 and HPV58, may be a relevant option for this region, and support enhanced follow-up for HPV52/58-positive women as well as integrated management of dysbiosis. By providing the most recent data (2019–2024, including the post-2022 stabilization phase) and the first genotype-specific, dose–response evidence on HPV-microecology from Chongqing, this study fills a critical gap in China’s cervical cancer surveillance.

## 1 Introduction

Malignant tumors are among the most common causes of death worldwide. GLOBOCAN 2022 data indicates that cervical cancer is the fourth most common cancer in women globally (1). It is the most common cancer of the female genital tract in developing countries, accounting for about 85% of new cases (2). According to the National Cancer Center, China has a high endemic situation, with about 106,000 new cases and 34,000 deaths reported annually, which corresponds to incidence and mortality rates of 7.6 and 2.4 per 100,000, respectively (3, 4).

A widely accepted understanding states that the persistent infection with high-risk human papillomavirus (HR-HPV) is required to cause cervical cancer (5). The majority of cervical cancer patients are positive for HR-HPV, and the virus is associated with cervical intraepithelial neoplasia (CIN) development (6). Thus, the control of HR-HPV infection is a key primary prevention strategy. Similarly, the HPV test among women aged 25–64 years is part of a secondary prevention activity that detects cancer early and treats it promptly (7). Although screening coverage has been expanded in China, large regional gaps still exist. According to the WHO, in the community populations of Southwest China, screening coverage is lower than 40%, and HPV vaccination is at a lower level, unevenly distributed, and complicating prevention efforts targeting specific regions (8). In addition, HPV genotypes tend to differ across regions, while multiple infections may play a synergistic role in neoplastic progression that needs regionally targeted prevention.

So far, researchers have identified more than 200 HPV genotypes and classified them into high-risk (HR) and low-risk (LR) types (9). PCR reverse hybridization has become the method of choice for large -scale epidemiological studies because of its accurate genotyping capability and broad subtype coverage (10, 11). HR types—particularly HPV16, 18, 31, 33, 45, 52, and 58—are the principal drivers of cervical, anal, and oropharyngeal cancers (12, 13). Worldwide, HPV16 and 18 together account for approximately 70% of invasive cervical cancers (14). LR types (e.g., HPV6, 11, 42, and 43) predominantly cause benign anogenital warts with minimal malignant potential (15).

Importantly, HPV prevalence and genotype distribution exhibit strong regional heterogeneity. In China, northern provinces show a predominance of HPV16 and 58, whereas southern and southwestern regions have significantly higher infection rates of HPV16, 52, and 53. As a major urban center in Southwest China, Chongqing has only limited data available: previous studies covered the period 2017–2022 and lacked a systematic assessment of how vaginal microecology interacts with HPV infection and cervical lesions. In particular, no large-scale study has investigated whether shifts in HPV epidemiology have occurred in Chongqing following the gradual rollout of HPV vaccination and expanded screening coverage in recent years. While a recent study in Chongqing reported an association between vaginal dysbiosis and overall HR HPV positivity (16), it did not examine genotype-specific patterns nor assess dose–response relationships across vaginal cleanliness grades. Therefore, whether HPV52 and HPV16 differ in their susceptibility to microecological disturbances remains unknown.

In contrast to prior cross-sectional and short-term studies, no large-scale research in Chongqing has spanned the post-screening implementation period (2019–2024) or systematically integrated vaginal microecology with genotype-specific HPV analysis. To address this gap, we conducted a six-year (2019–2024) hospital -based epidemiological study enrolling 24,050 eligible women at the People’s Hospital of Liangjiang New Area, Chongqing. Using PCR reverse dot blot hybridization to detect 23 HPV subtypes, we systematically evaluated: (1) the distribution of HPV genotypes and age specific infection patterns; (2) associations between HPV infection and abnormal cervical cytology; and (3) the relationship between HPV genotype profiles and vaginal microecological status. This study provides three distinct contributions: (1) it provides the most recent continuous HPV surveillance (2019–2024) from Chongqing to date; (2) it provides the first genotype-specific, dose–response characterization of HPV microecology associations in this region; and (3) it offers updated local evidence to inform next generation multivalent vaccines targeting regionally prevalent genotypes such as HPV52 and HPV53. These findings are expected to help improve screening protocols, guide vaccination, and support precision prevention efforts for cervical cancer in Southwest China.

## 2 Materials and methods

### 2.1 Study design

Data for this retrospective observational study were collected at the People‘s Hospital of Liangjiang New Area, Chongqing, covering a six-year period from January 1, 2019, to December 31, 2024. This is a repeated cross-sectional study; individual women were not followed longitudinally. The study protocol was approved by the Institutional Ethics Committee of the People’s Hospital of Liangjiang New Area. The study was conducted at the People’s Hospital of Chongqing Liangjiang New Area, a grade A tertiary hospital serving both the local community and surrounding districts. The hospital provides routine gynecological care, cervical cancer screening, and referral services.

### 2.2 Study population

**Figure 1:**
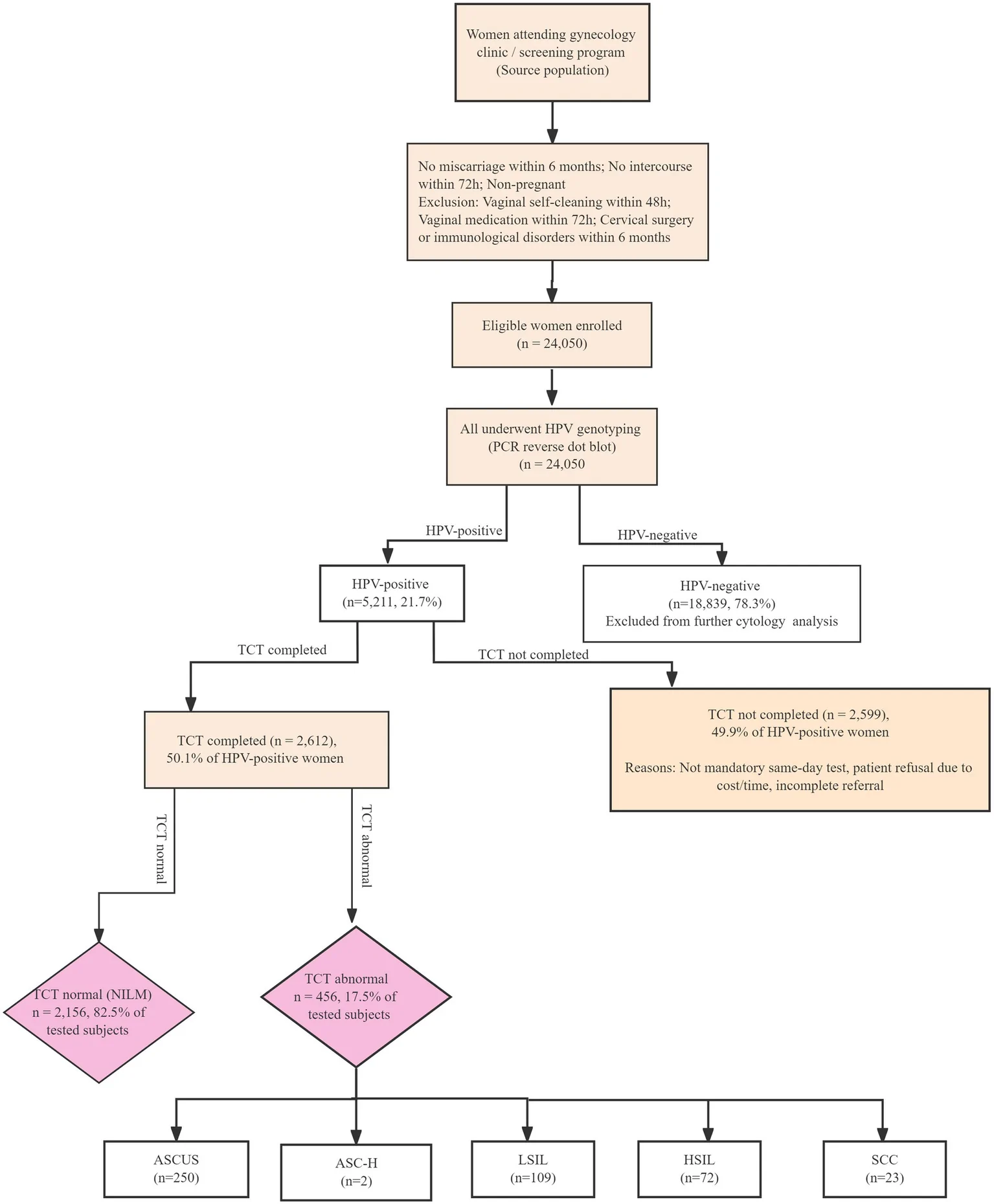
A flowchart detailing patient inclusion and exclusion for each analysis step.

A total of 24,050 eligible female patients who attended the hospital during the study period were enrolled (Figure 1). Inclusion criteria were as follows: (1) no history of miscarriage within the preceding 6 months; (2) no sexual intercourse within 72 h before sample collection; and (3) non-pregnant status. Exclusion criteria were as follows: (1) self-cleaning of the external genitalia within 48 h before examination; (2) use of any vaginal medication within 72 h before sample collection; (3) history of cervical surgery or immunological disorders within the past 6 months. The exclusion of women with recent miscarriage (within 6 months) was to avoid potential confounding from pregnancy-related hormonal changes and cervical remodeling, which could affect HPV detection and cytology results. The exclusion of women with recent sexual intercourse (within 72 h) and vaginal medication use (within 72 h) was to minimize sample contamination and ensure specimen quality. All women who attended the hospital’s gynecology outpatient clinic, health examination center, or cervical cancer screening program during the study period and met eligibility criteria were consecutively enrolled. Participants aged 18 to 80 years and were stratified into six groups for analysis: ≤20, 21–30, 31–40, 41–50, 51–60, and >60 years.

### 2.3 Cervical sample collection

Cervical epithelial cells were collected by experienced gynecologists. Residual secretions around the external cervical os were first gently removed using a sterile cotton swab. A disposable cervical cell brush was then inserted into the cervical os and rotated clockwise or counterclockwise at least three full turns. Samples were stored at 4 °C or −20 °C.

### 2.4 DNA extraction

Genomic DNA was extracted via the thermal lysis method. Briefly, 500 μL of the cervical cell preservation solution was transferred to a 1.5 mL sterile centrifuge tube and centrifuged at 13,000 rpm for 10 min. The supernatant was discarded. The cell pellet was resuspended in 100 μL of lysis buffer. The tube was incubated in a boiling water bath for 10 min, then cooled on ice for 10 min, and centrifuged again at 13,000 rpm for 10 min. The supernatant was collected as the DNA template. The extracted DNA was stored at 4 °C for short-term within 7 days, or at −20 °C for long term storage.

### 2.5 HPV genotyping

HPV genotyping was performed using the Human Papillomavirus 23 Genotype Detection Kit (Cat. No. YN HPV 23, Yaneng Biotechnology, China), which detects 6 low-risk types (HPV6, 11, 42, 43, 81, 83) and 17 high-risk genotypes (HPV16, 18, 58, 52, 31, 33, 59, 45, 51, 56, 73, 66, 39, 53, 68, 82, 35). The HPV genotyping kit (YN HPV 23, Yaneng Biotechnology) has been validated in previous studies with reported sensitivity of >95% and specificity of >98% for the 23 genotypes included. Each assay run included a negative control (sterile water) and a positive control (plasmid DNA containing HPV-16 type) to monitor for contamination and amplification efficiency. Inter-run and intra-run reproducibility was assessed by repeating 5% of samples in duplicate, with concordance rates >99%. High-risk classification followed the 2022 International Agency for Research on Cancer (IARC) criteria, with HPV53 and 66 designated as probable high-risk genotypes. PCR amplification was carried out on an ABI7500 system (Life Technologies, USA). After amplification, PCR products were denatured and chilled. Membrane hybridization, post-hybridization washing, incubation with streptavidin horseradish peroxidase conjugate, and colorimetric detection were performed strictly. Every assay included a negative control and a positive control.

### 2.6 ThinPrep cytology test

All cervical samples were subjected to TCT in the Department of Pathology. To minimize diagnostic bias, two senior pathologists independently evaluated each specimen. In cases of diagnostic disagreement (<5% of samples), a third senior pathologist was consulted, and the majority opinion was followed.

Cytological diagnoses were classified according to the Bethesda System 2014 into six categories: negative for intraepithelial lesion or malignancy (NILM); atypical squamous cells of undetermined significance (ASCUS); atypical squamous cells, cannot exclude high grade squamous intraepithelial lesion (ASC H); low grade squamous intraepithelial lesion (LSIL); high grade squamous intraepithelial lesion (HSIL); and squamous cell carcinoma (SCC).

TCT was not uniformly performed on all HPV-positive women for several reasons: (a) clinical practice at our hospital does not mandate same-day TCT for all HPV-positive patients without specific indications (e.g., abnormal bleeding, visible lesions); (b) some patients declined same-day testing due to financial constraints, opting to schedule it separately; and (c) some patients were referred for TCT but did not complete the referral within the study period. This resulted in 2,612 of 5,211 (50.1%) HPV-positive women undergoing TCT.

### 2.7 Vaginal microecology assessment

Vaginal secretions were collected from the upper third of the vaginal wall using two sterile cotton swabs. The secretions were fully eluted by repeated stirring and squeezing. An automated gynecological secretion analyzer (Model GMD S600, Dirui, China) coupled with matched test strips was applied for dry chemical detection of vulvovaginal candidiasis (VVC), bacterial vaginosis (BV) and trichomonal vaginitis (TV), and. Wet mount microscopy was performed as the gold standard for detecting fungal hyphae and spores as well as motile trichomonads.

Vaginal microecology was classified into four categories: BV, TV, VVC, and normal control (NC). Samples with concurrent BV and VVC (or other mixed infections) were categorized as mixed infection and excluded from subgroup analyses of single infection. Vaginal cleanliness was graded according to the Chinese Society of Obstetrics and Gynecology (2020) guidelines: Grade I—predominant lactobacilli, white blood cells (WBC) < 5 per high power field (HPF), abundant epithelial cells, no miscellaneous bacteria; Grade II—reduced lactobacilli, WBC 5–15/HPF, moderate epithelial cells, few miscellaneous bacteria; Grade III—scarce lactobacilli, WBC 15–30/HPF, few epithelial cells, moderate to many miscellaneous bacteria; Grade IV—absent lactobacilli, WBC > 30/HPF, rare epithelial cells, massive miscellaneous bacteria. Grades I and II were defined as normal. Grades III and IV indicated vaginal microecological imbalance.

### 2.8 Statistical analysis

Statistical analyses were performed using R software, Python and SPSS 25.0. HPV positivity rates were presented as percentages. Enumeration data were used to summarize positive and negative cases per HPV genotype. Intergroup comparisons were performed with chi-square tests and t-test. Heatmaps visualized HPV prevalence across disease categories and age groups. Origin 2021 (OriginLab Corp., USA) and GraphPad Prism 6 (USA) were used for all data. Statistical significance was set at *p* < 0.05. To enable comparisons across years and age groups, Z-score normalization was performed for each genotype across different years (Figure 1A) and different age groups (Figure 1B).

## 3 Results

### 3.1 Overall HPV prevalence and temporal trends

**Figure 2:**
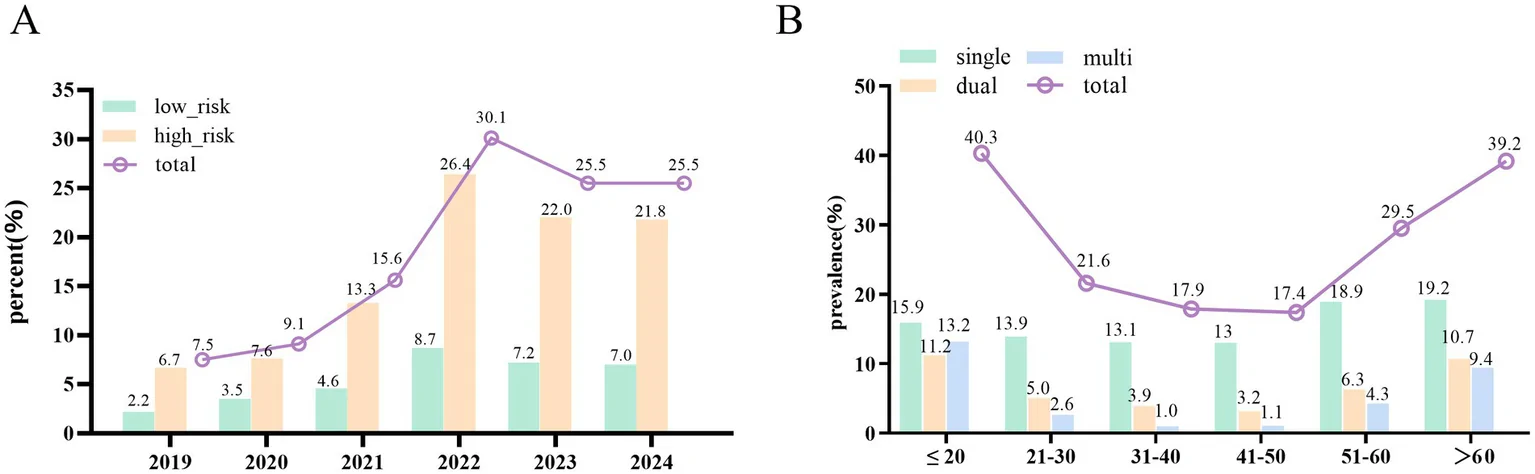
Prevalence of HPV infection from 2019 to 2024. **(A)** The prevalence of low-risk and HR-HPV types from 2019 to 2024. **(B)** Distribution of single and multiple HPV infections across different age groups. *n* = 24,050.

From 2019 to 2024, a total of 24,050 eligible female patients were enrolled, among whom 5,211 tested positive for HPV, yielding an overall pooled positivity rate of 21.7% (5,211/24,050). The annual HPV positivity rates showed a significant increasing trend from 2019 to 2022 (7.5% [105/1,397] in 2019, 9.1% [272/3,005] in 2020, 15.6% [600/3,838] in 2021, and 30.1% [1,315/4,368] in 2022), followed by stabilization in 2023 (25.5%, 1,485/5,818) and 2024 (25.5%, 1,434/5,624). The Cochran Armitage trend test confirmed a significant upward trend from 2019 to 2022 (*p* < 0.001) and no further change for 2023–2024 (Figure 2A).

High-risk HPV (HR-HPV) positivity was consistently higher than low-risk HPV (LR-HPV) positivity in each calendar year (all *p* < 0.001). Overall, HR HPV accounted for 86.2% (4,493/5,211) of all positive cases, while LR HPV accounted for the remaining 29.0% (1,510/5,211). These percentages exceed 100% because some women were co-infected with both HR and LR genotypes. Among the 5,211 HPV positive patients, 3,475/5211 (66.7%) had single genotype infections, 1,157/5211 (22.2%) had double infections, 361/5211 (6.9%) had triple infections, and 218/5211 (4.2%) had quadruple or more infections (Table 1).

**Table 1:** Distribution of age, cytology findings, and HPV infection in the study population.

Characteristics	N	%
Age group(year)		
≤20	454	1.9%
21–30	8,689	36.1%
31–40	7,897	32.8%
41–50	4,050	16.8%
51–60	2,323	9.7%
>60	637	2.7%
Cytology		
NLIM	2,156	82.5%
ASCUS	250	9.6%
ASC-H	2	0.1%
LSIL	109	4.2%
HSIL	72	2.8%
SCC	23	0.9%
HPV prevalence (overall)	5,211	21.7%
Single infection	3,475	66.7%
dual infections	1,157	22.2%
triple infections	361	6.9%
quadruple or more infections	218	4.2%
HR-HPV^a^	4,493	18.7%
LR-HPV^b^	1,510	6.3%

HR-HPV, high-risk HPV types (16, 18, 58, 52, 31, 33, 59, 45, 51, 56, 73, 66, 39, 53, 68, 82, 35); LR-HPV, low-risk HPV types (6, 11, 42, 43, 81, 83); HPV, human papillomavirus.

The age distribution of the study population was as follows: ≤20 years, 454/24050 (1.9%); 21–30 years, 8,689/24050 (36.1%); 31–40 years, 7,897/24050 (32.8%); 41–50 years, 4,050/24050 (16.8%); 51–60 years, 2,323/24050 (9.7%); and >60 years, 637/24050 (2.7%; Table 1). The HPV positivity rate varied significantly across age groups (*p* < 0.001), exhibiting a bimodal pattern. The highest positivity rates were observed in the ≤20 years (40.3%, 183/454) and the >60 years (39.2%, 250/637), followed by the 51–60 years (29.5%, 685/2,323; Figure 2B).

### 3.2 Genotype distribution of single and multiple HPV infections

**Figure 3:**
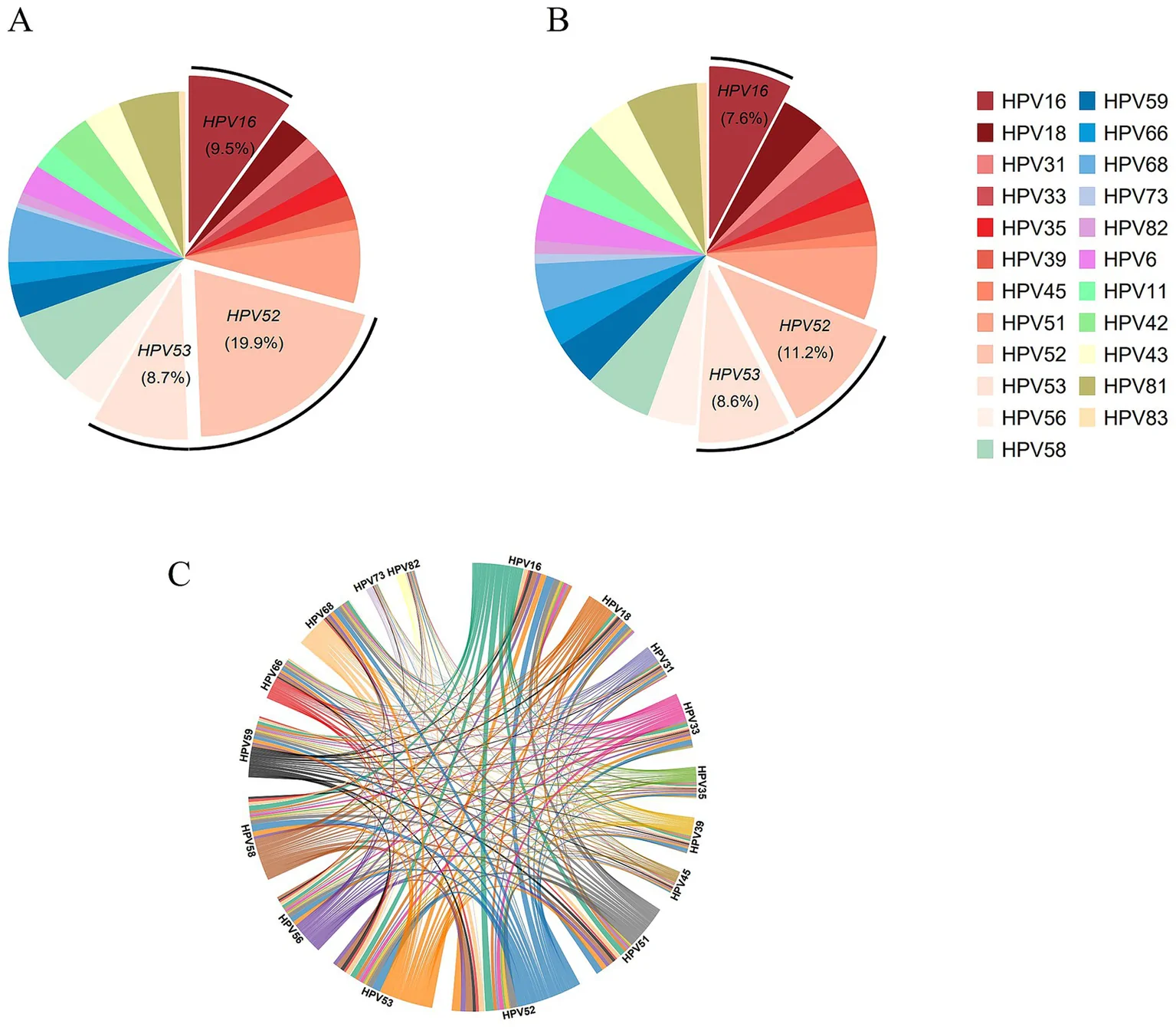
Distribution of different types of HPV infection. Genotype distribution of single (*n* = 3,475) **(A)** and multiple (*n* = 4,418) HPV infections **(B)**. Node diagram of different type paired combinations in HPV multiple infection patients (*n* = 2,788) **(C)**.

Among single infections, HR-HPV was the predominant type (Figure 3A). HPV52 was the most prevalent genotype (19.9%, 690/3475), followed by HPV16 (9.5%, 329/3475) and HPV53 (8.7%, 304/3475). Among multiple infections, HR-HPV remained predominant (Figure 3B). HPV52 was again the most common genotype (11.2%, 493/4418), followed by HPV53 (8.6%, 379/4418) and HPV16 (7.6%, 337/4418). LR-HPV types constituted a smaller proportion, with HPV81 and HPV6 being the most frequent.

The most frequent pairwise combinations were HPV52 and HPV53 (79 times), HPV16 + HPV52 (75 times), and HV52 + HPV58 (64 times). The chord diagram visualizes these HR HPV dominant pairing patterns (Figure 3C).

### 3.3 Temporal and age specific patterns of HPV infection

**Figure 4:**
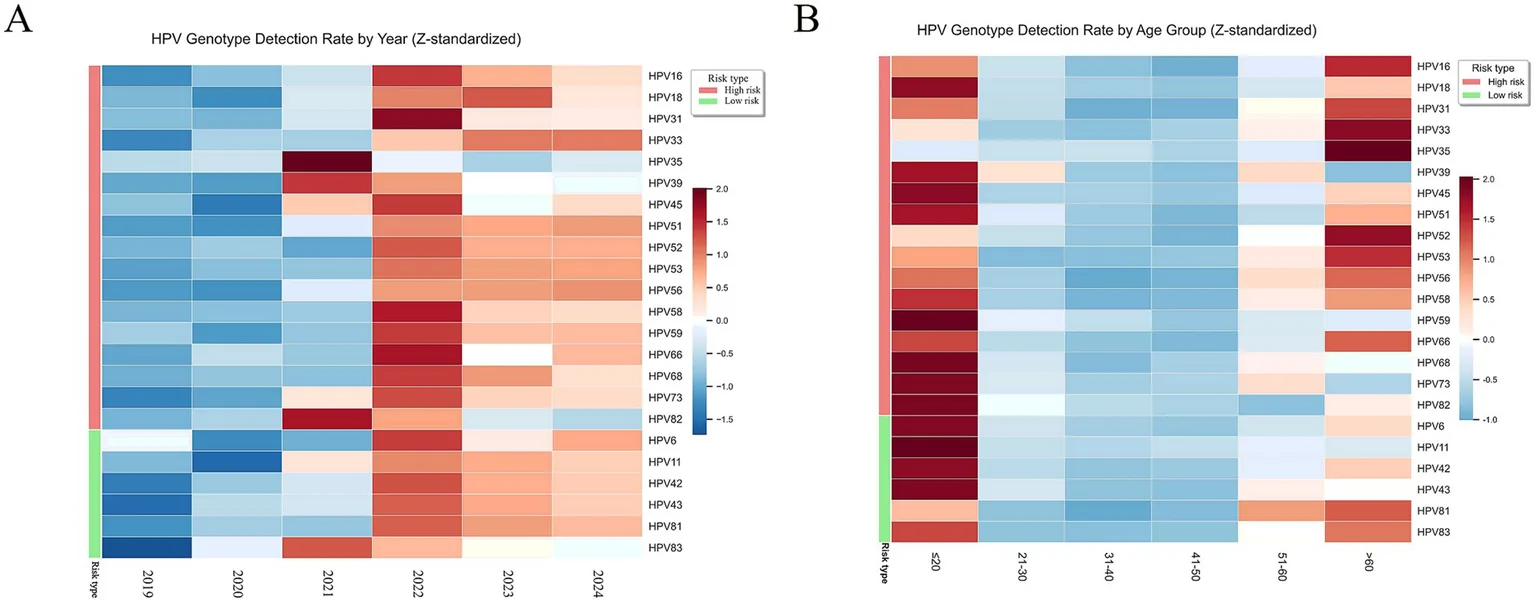
Age-specific and year-specific prevalence of HPV infection. **(A)** Heatmap of HPV genotype detection rates in different age groups. **(B)** Heatmap of HPV genotype detection rates in different year groups. Z-score normalization was performed for each genotype across different years **(A)** and age groups **(B)**. For a given genotype, *x* represents its raw detection rate in a specific year or age group, *μ* is the mean detection rate of that genotype across all years or age groups, and *σ* is the standard deviation of its detection rates across all years or age groups. The normalized *Z*-score was calculated as *Z* = (*x* − *μ*)/*σ*. *n* = 5,211.

Figure 4A shows that HPV52 was the most consistently detected genotype from 2019 to 2024, with detection proportions of 12.1 to 27.2% among all positive cases. HPV53 was the second most frequently detected HR-HPV in most years. The proportions of HPV16 and HPV58 decreased modestly over time (HPV16: from 14.3% in 2019 to 11.2% in 2024; HPV58: from 12.8% in 2022 to 9.4% in 2024). The absolute number of HR-HPV positive cases increased from 93 in 2019 to a peak of 1,280 in 2023, followed by a slight decline to 1,226 in 2024; LR-HPV positive cases increased from 31 in 2019 to a peak of 421 in 2023, then declined to 394 in 2024 (Table 2).

**Table 2:** Distribution of HPV infection by year.

HPV subtypes	2019	2020	2021	2022	2023	2024
HR	93	229	512	1,153	1,280	1,226
HPV16	15 (14.3%)	45(16.5%)	75(14.7%)	176(13.4%)	188(12.7%)	160(11.2%)
HPV18	8 (7.6%)	12(4.4%)	33(6.5%)	66(5.0%)	95(6.4%)	64(4.5%)
HPV31	4 (3.2%)	8(2.9%)	17(3.3%)	49(3.7%)	36(2.4%)	34(2.4%)
HPV33	3 (2.9%)	17(6.3%)	21(4.1%)	51(3.9%)	83(5.6%)	80(5.6%)
HPV35	6 (5.7%)	15(5.5%)	77(15.1%)	30(2.3%)	21(1.4%)	33(2.3%)
HPV39	6 (5.7%)	12(4.4%)	48(9.4%)	46(3.5%)	45(3.0%)	42(2.9%)
HPV45	3 (2.9%)	3(1.1%)	18(3.5%)	28(2.1%)	21(1.4%)	25(1.8%)
HPV51	8 (7.6%)	14(5.2%)	61(11.9%)	130(9.9%)	161(10.9%)	162(11.4%)
HPV52	27 (25.7%)	74(27.2%)	62(12.1%)	318(24.2%)	352(23.8%)	339(23.8%)
HPV53	9 (8.6%)	31(11.4%)	42(8.2%)	176(13.4%)	209(14.1%)	200(14.0%)
HPV56	7 (6.7%)	14(5.2%)	40(7.8%)	74(5.6%)	99(6.7%)	98(6.9%)
HPV58	11 (10.5%)	26(9.6%)	39(7.6%)	169(12.8%)	144(9.7%)	134(9.4%)
HPV59	8 (7.6%)	8(2.9%)	20(3.9%)	90(6.8%)	86(5.8%)	85(%6.0)
HPV66	3 (2.9%)	16(5.9%)	15(2.9%)	77(5.9%)	47(3.2%)	67(4.7%)
HPV68	8 (7.6%)	21(7.7%)	26(5.1%)	110(8.4%)	122(8.2%)	92(6.5%)
HPV73	1 (1.0%)	3(1.1%)	9(1.8%)	15(1.1%)	15(1.0%)	14(1.0%)
HPV82	1 (1.0%)	5(1.8%)	33(6.5%)	26(2.0%)	15(1.0%)	10(0.7%)
LR	31	105	177	382	421	394
HPV6	15 (14.3%)	15 (5.5%)	25(4.9%)	79(6.0%)	70(4.7%)	84(5.9%)
HPV11	9 (8.6%)	14 (5.2%)	36(7.1%)	49(3.7%)	62(4.2%)	56(3.9%)
HPV42	1(1.0%)	17 (6.3%)	32(6.3%)	89(6.8%)	95(6.4%)	83(5.8%)
HPV43	3 (2.9%)	23(8.5%)	33(6.5%)	75(5.7%)	86(5.8%)	75(5.3%)
HPV81	6 (5.7%)	29(10.7%)	34(6.7%)	130(9.9%)	151(10.2%)	134(9.4%)
HPV83	0(0.0%)	5(1.8%)	12(2.4%)	11(0.8%)	11(0.7%)	10(0.7%)

Age stratified analysis revealed distinct age dependent preferences (Figure 4B). LR-HPV types 6 and 11 were significantly more prevalent in the ≤20 years than the overall average (*p* < 0.05). HR-HPV types 16, 52, and 53 peaked in the 41–50 years and remained elevated in the 51–60 years. HPV52 exhibited cross age dominance, with high positivity rates in the ≤20 year, 41–50 years, and >60 years. LR-HPV83 showed uniformly low prevalence across all ages.

### 3.4 Association between HPV infection and cervical lesions

**Figure 5:**
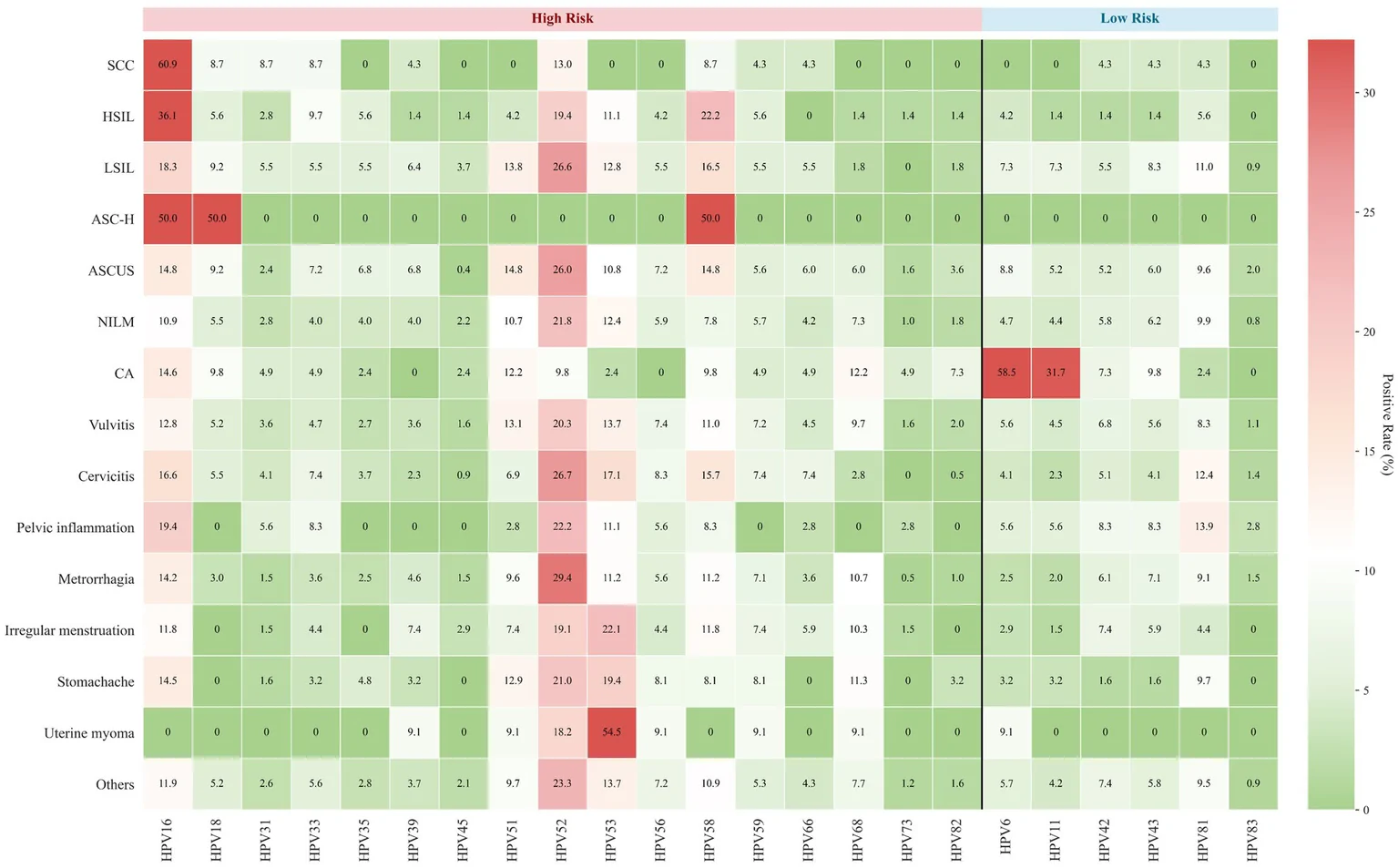
HPV type-specific positivity rates by disease group in Chongqing, China, 2019–2024. *n* = 5,211.

Among the 5,211 HPV-positive patients, 2,612 underwent TCT. Of these, 2,156/2612 (82.6%) had normal cytology (NILM), and 456/2612 (17.5%) showed abnormal cytology, including 250 cases of ASCUS, 2 of ASC-H, 109 of LSIL, 72 of HSIL, and 23 of SCC (Table 1). As depicted in Figure 5, HPV16 displayed a distinct gradient relative to lesion severity: 18.3% (20/109) in LSIL, 36.1% (26/72) in HSIL and 60.9% (14/23) in SCC identifying it as a driver of malignant progression. In LSIL (26.6%), HPV52 was the most common genotype. It was also the second most frequent in other categories such as ASCUS (26.0%). Moreover, HPV52 also ranked highly in other non-cervical gynecological conditions such as Vulvitis (20.3%) and abnormal uterine bleeding (29.4%). Another common category was abdominal pain (21.0%). In case of condyloma acuminata (CA), LR-HPV types were dominant with HPV6 (58.5%) and HPV11 (31.7%) significantly more common than other types (*p* < 0.01).

Beyond genotype-specific associations, the impact of infection multiplicity was also examined. Comparisons between single and multiple infections by genotype were presented in Table 3. Overall, the proportion of abnormal cytology (ASCUS, LSIL, HSIL, and SCC combined) was higher in women with multiple infections than in those with single infections. This pattern was most evident for HPV6 and HPV11, where multiple infections were associated with higher proportions of LSIL/ASCUS compared with single infections.

**Table 3:** Association of single and multiple HPV infections with cervical lesion.

HPV type	N	ASCUS(%)	LSIL(%)	HSIL(%)	SCC(%)
Single_HPV16	329	15(4.6)	6(1.8)	13(4.0)	10(3.0)
Multiple_HPV16	337	22(6.5)	14(4.2)	13(3.9)	4(1.2)
Single_HPV18	98	8(8.2)	2(2.0)	2(2.0)	1(1.0)
Multiple_HPV18	184	15(8.2)	8(4.3)	2(1.1)	1(0.5)
Single_HPV31	52	1(1.9)	1(1.9)	1(1.9)	1(1.9)
Multiple_HPV31	98	5(5.1)	5(5.1)	1(1.0)	1(1.0)
Single_HPV33	97	4(4.1)	1(1.0)	3(3.1)	2(2.1)
Multiple_HPV33	163	14(8.8)	5(3.1)	4(2.5)	0(0.0)
Single_HPV35	81	5(6.2)	0(0.0)	2(2.5)	0(0.0)
Multiple_HPV35	105	12(11.4)	6(5.7)	2(1.9)	0(0.0)
Single_HPV39	84	3(3.6)	3(3.6)	0(0.0)	1(1.2)
Multiple_HPV39	124	14(11.3)	4(3.2)	1(0.8)	0(0.0)
Single_HPV45	36	0(0.0)	2(5.6)	0(0.0)	0(0.0)
Multiple_HPV45	65	1(1.5)	2(3.1)	1(1.5)	0(0.0)
Single_HPV51	241	9(3.7)	5(2.1)	0(0.0)	0(0.0)
Multiple_HPV51	303	28(9.2)	10(3.3)	3(1.0)	0(0.0)
Single_HPV52	690	30(4.3)	17(2.5)	8(1.2)	1(0.1)
Multiple_HPV52	493	35(7.1)	12(2.4)	6(1.2)	2(0.4)
Single_HPV53	304	3(1.0)	6(2.0)	2(0.7)	0(0.0)
Multiple_HPV53	379	24(6.4)	8(2.1)	6(1.6)	0(0.0)
Single_HPV56	132	5(3.8)	0(0.0)	0(0.0)	0(0.0)
Multiple_HPV56	206	13(6.3)	6(2.9)	3(1.5)	0(0.0)
Single_HPV58	254	15(5.9)	5(2.0)	10(3.9)	1(0.4)
Multiple_HPV58	279	22(7.9)	13(4.7)	6(2.2)	1(0.4)
Single_HPV59	111	3(2.7)	1(0.9)	1(0.9)	0(0.0)
Multiple_HPV59	193	11(5.7)	5(2.6)	3(1.6)	1(0.5)
Single_HPV66	77	4(5.2)	1(1.3)	0(0.0)	0(0.0)
Multiple_HPV66	151	11(7.3)	5(3.3)	0(0.0)	1(0.7)
Single_HPV68	183	7(3.8)	0(0.0)	1(0.5)	0(0.0)
Multiple_HPV68	200	8(4.0)	2(1.0)	0(0.0)	0(0.0)
Single_HPV73	16	1(6.2)	0(0.0)	1(6.2)	0(0.0)
Multiple_HPV73	41	3(7.3)	0(0.0)	0(0.0)	0(0.0)
Single_HPV82	38	2(5.3)	0(0.0)	1(2.6)	0(0.0)
Multiple_HPV82	54	7(13.0)	2(3.7)	0(0.0)	0(0.0)
Single_HPV6	98	14(14.3)	1(1.0)	2(2.0)	0(0.0)
Multiple_HPV6	192	35(18.2)	13(6.8)	2(1.0)	1(0.5)
Single_HPV11	86	2(2.3)	1(1.2)	0(0.0)	0(0.0)
Multiple_HPV11	141	11(7.8)	7(5.0)	1(0.7)	0(0.0)
Single_HPV42	133	3(2.3)	0(0.0)	0(0.0)	0(0.0)
Multiple_HPV42	191	10(5.2)	6(3.1)	1(0.5)	1(0.5)
Single_HPV43	125	2(1.6)	2(1.6)	1(0.8)	0(0.0)
Multiple_HPV43	183	13(7.1)	7(3.8)	0(0.0)	1(0.5)
Single_HPV81	195	6(3.1)	1(0.5)	0(0.0)	0(0.0)
Multiple_HPV81	301	18(6.0)	11(3.7)	4(1.3)	1(0.3)
Single_HPV83	15	1(6.7)	0(0.0)	0(0.0)	0(0.0)
Multiple_HPV83	35	4(11.4)	1(2.9)	0(0.0)	0(0.0)

### 3.5 Age distribution of cervical lesions

**Figure 6:**
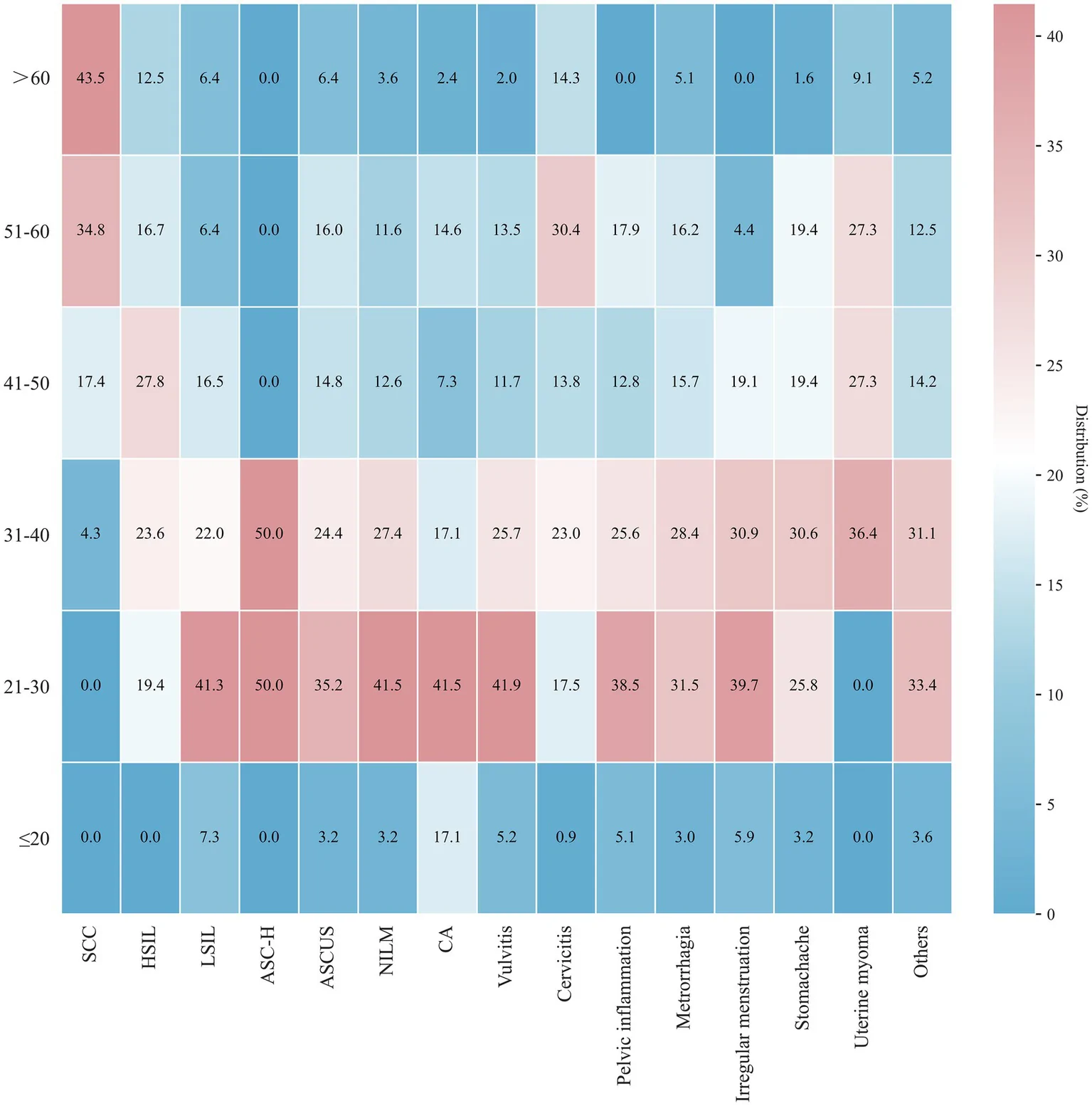
The overall HPV positive rate by disease group and age group in Chongqing, China from 2019 to 2024.

The composition of cervical lesions differed significantly across age groups (*p* < 0.001, Figure 6). The 21–30 years had the highest prevalence of several lesion types: ASC-H 50.0%, vulvitis (41.9%), CA (41.5%), and LSIL (41.3%). HSIL was most common in the 41–50 years (27.8%), followed by the 31–40 years (23.6%). SCC was concentrated in older age groups: (43.5%) in the >60 years and (34.8%) in the 51–60 years.

### 3.6 Genotype specific distribution across age groups and lesion types

Among the 456 patients with abnormal cytology, TCT positivity rates differed significantly by age (*p* < 0.05), with the highest rate in the >60 years (35.0%), followed by the 41–50 years (22.6%), and the lowest in the 21–30 years (14.2%; Table 4).

**Table 4:** TCT positive rates of different ages.

Age(years)	N	NLIM, n (%)	ASCUS, n(%)	ASC-H, n (%)	LSIL, n (%)	HSIL, n (%)	SCC, n (%)	TCT positive (%)
≤20	86	70(81.4%)	8(9.3%)	0(0.0%)	8(9.3%)	0(0.0%)	0(0.0%)	16(18.6%)
21–30	1,043	895(85.8%)	88(8.4%)	1(0.1%)	45(4.3%)	14(1.3%)	0(0.0%)	148(14.2%)
31–40	695	591(85.0%)	61(8.8%)	1(0.1%)	24(3.5%)	17(2.5%)	1(0.1%)	104(15.0%)
41–50	350	271(77.4%)	37(10.6%)	0(0.0%)	18(5.1%)	20(5.7%)	4(1.1%)	79(22.6%)
51–60	318	251(78.9%)	40(12.6%)	0(0.0%)	7(2.2%)	12(3.8%)	8(2.5%)	67(21.1%)
>60	120	78(65.00%)	16(13.3%3)	0(0.0%)	7(5.8%)	9(7.5%)	10(8.3%)	42(35.0%)
Total	2,612	2,156	250	2	109	72	23	456

**Figure 7:**
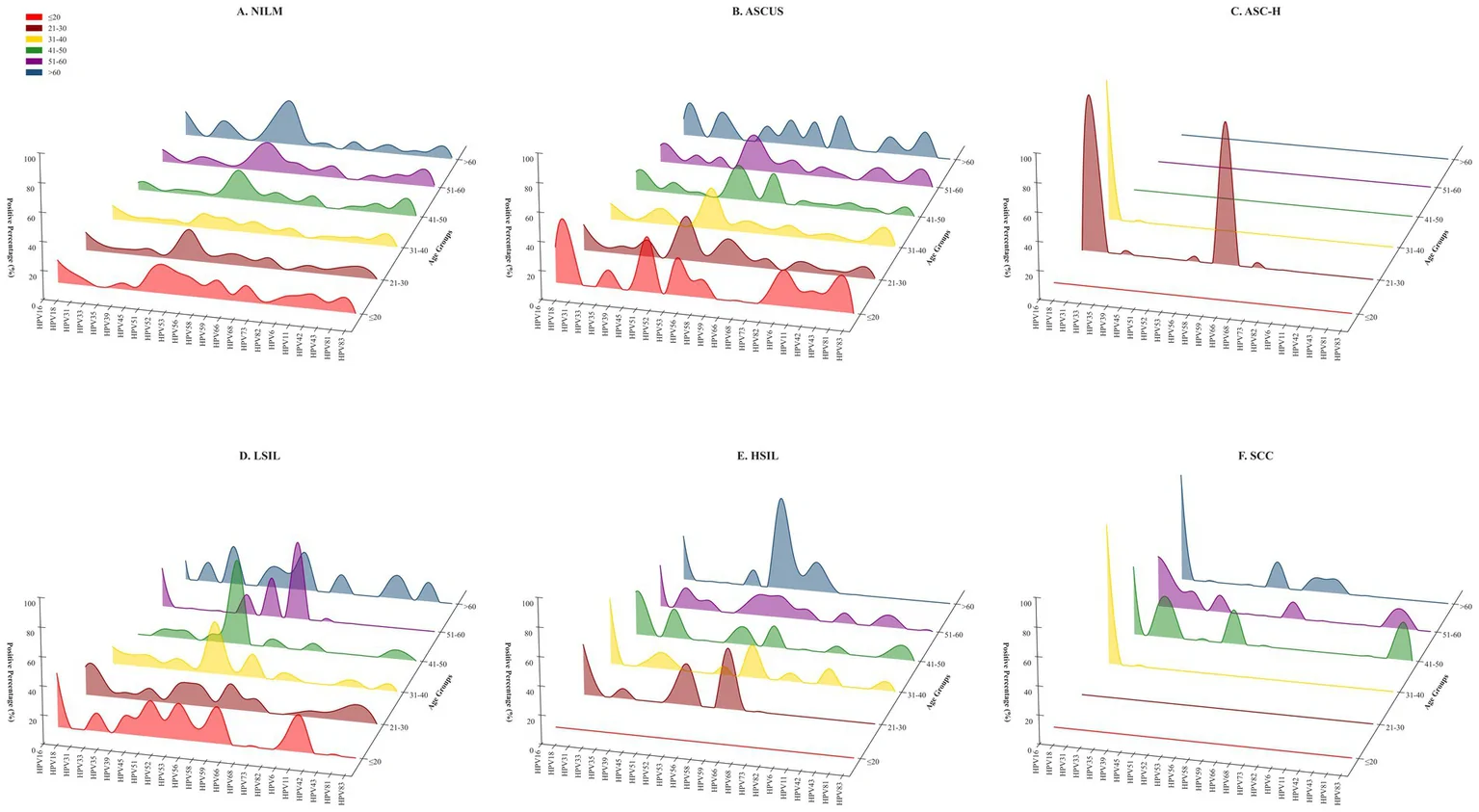
HPV type-specific positivity rates in Chongqing, China (2019-2024). **(A)** NLIM (*n* = 2,156); **(B)** ASCUS (*n* = 250). **(C)** ASC-H (*n* = 2). **(D)** LSIL (*n* = 109); **(E)** HSIL (*n* = 72); **(F)** SCC (*n* = 23).

Age-specific genotype distributions were observed for high-grade lesions. In HSIL, HPV16 positivity was highest in the 31–40 years (47.1%, 8/17), followed by the 21–30 years (35.7%, 5/14), and the 41–50 years (31.6%, 6/19). Notably, among women >60 years, HPV52 positivity in HSIL reached 66.7% (6/9), becoming the dominant genotype in this age stratum. In SCC, HPV16 positivity was 100% in the 31–40 years, 50.0% in the 41–50 years, and 37.5% in the 51–60 years, confirming its role as the core carcinogenic genotype in adults aged 31–60 years. HPV18, 31, 33, 52, 58 and 81 also contributed to SCC across age groups (Figure 7).

Overall, HR-HPV was detected in 421 of 456 cervical lesions (92.3%). Among these, HPV16 was the most common (21.5%, 98/456), rising to 60.9% (14/23) in SCC. HPV52 (24.3%, 111/456), HPV58 (16.2%, 74/456), and HPV53 (10.7%, 49/456) were also frequently detected. In SCC, HPV52 accounted for 13.0% (3/23) and HPV58 for 8.7% (2/23), making them the most prevalent HR-HPV types after HPV16. HPV6 was detected in some lesions but appeared exclusively in the context of multiple infections. Importantly, no cervical lesion (CIN or carcinoma) was attributable to LR-HPV alone in the absence of HR-HPV co-infection (Table 5).

**Table 5:** Detection rate of HPV genotypes in cervical precancerous lesions and cervical cancer.

HPV type	Risk for cancer	ASCUS, ASC-H, LSIL, HSIL, SCC (n/N%), *N* = 456	Single-infected (n/N%), *N* = 456	Multiple-infected (n/N%), *N* = 456	SCC (n/N%), *N* = 23	Single-infected in SCC (n/N%), *N* = 23
16	H	98(21.5%)	45(9.9%)	53(11.6%)	14(60.9%)	10(43.5%)
18	H	40(8.8%)	13(2.9%)	27(5.9%)	2(8.7%)	1(4.3%)
31	H	16(3.5%)	4(0.9%)	12(2.6%)	2(8.7%)	1(4.3%)
33	H	33(7.2%)	10(2.2%)	23(5.0%)	2(8.7%)	2(8.7%)
35	H	27(5.9%)	7(1.5%)	20(4.4%)	0(0.0%)	0(0.0%)
39	H	26(5.7%)	7(1.5%)	19(4.2%)	1(4.3%)	1(4.3%)
45	H	6(1.3%)	2(0.4%)	4(0.9%)	0(0.0%)	0(0.0%)
51	H	55(12.1%)	14(3.1%)	41(9.0%)	0(0.0%)	0(0.0%)
52	H	111(24.3%)	56(12.3%)	55(12.1%)	3(13.0%)	1(4.3%)
53	H	49(10.7%)	11(2.4%)	38(8.3%)	0(0.0%)	0(0.0%)
56	H	27(5.9%)	5(1.1%)	22(4.8%)	0(0.0%)	0(0.0%)
58	H	74(16.2%)	31(6.8%)	43(9.4%)	2(8.7%)	1(4.3%)
59	H	25(5.5%)	5(1.1%)	20(4.4%)	1(4.3%)	0(0.0%)
66	H	22(4.8%)	5(1.1%)	17(3.7%)	1(4.3%)	0(0.0%)
68	H	18(3.9%)	8(1.8%)	10(2.2%)	0(0.0%)	0(0.0%)
73	H	5(1.1%)	2(0.4%)	3(0.7%)	0(0.0%)	0(0.0%)
82	H	12(2.6%)	3(0.7%)	9(2.0%)	0(0.0%)	0(0.0%)
6	L	163(35.7%)	4(0.9%)	159(34.9%)	14(60.9%)	0(0.0%)
11	L	22(4.8%)	3(0.7%)	19(4.2%)	0(0.0%)	0(0.0%)
42	L	21(4.6%)	3(0.7%)	18(3.9%)	1(4.3%)	0(0.0%)
43	L	26(5.7%)	5(1.1%)	21(4.6%)	1(4.3%)	0(0.0%)
81	L	41(9.0%)	7(1.5%)	34(7.5%)	1(4.3%)	0(0.0%)
83	L	6(1.3%)	1(0.2%)	5(1.1%)	0(0.0%)	0(0.0%)

### 3.7 Association of HPV infection with vaginal microecology and cleanliness

**Figure 8:**
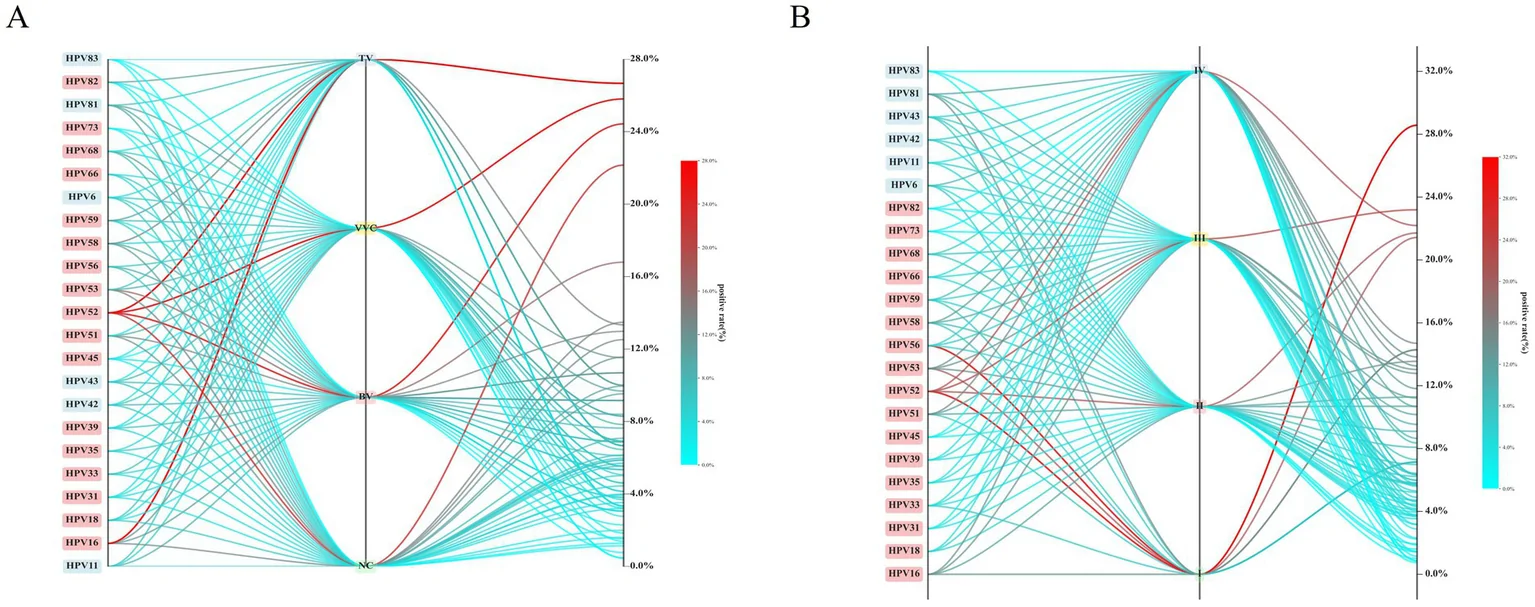
The positive rates of HPV types in relation to different vaginal microbiota outcomes (*n* = 3190) **(A)** and varying cleanliness levels (*n* = 3153) **(B)** in the Chongqing region of China from 2019 to 2024.

Vaginal microecology and cleanliness assessments were performed. Compared with the normal control (NC) group (defined as absence of BV, TV, and VVC with vaginal cleanliness Grade I and II), positivity rates of HPV52 and HPV16 were higher in the BV, TV, and VVC groups (*p* < 0.001, Figure 8A). In the TV group, HPV52 and HPV16 positivity both reached 26.7%. In the VVC group, HPV52 positivity was the highest (25.8%). In the BV group, HPV52 positivity was 24.4%.

HPV52 showed the highest positivity rate in Grade III (23.2%), whereas HPV16 was enriched in Grade IV (13.2%) and III (13.0%) compared with other grades, indicating a strong association with severe dysbiosis. The proportion of HR-HPV positivity increased progressively with worsening cleanliness grade (Cochran Armitage trend test, *p* < 0.001), suggesting that vaginal microecological imbalance is associated with HR-HPV positivity (Figure 8B).

### 3.8 Multivariable logistic regression analyses

To further evaluate the independent associations between HPV genotypes, vaginal microecology, and cervical lesions while adjusting for potential confounders, we performed multivariable logistic regression analyses. As shown in Supplementary Table S1, after adjusting for age, calendar year, and microecological abnormality, HPV16 (OR = 15.3, 95% CI: 3.4–68.5) and HPV58 (OR = 9.9, 95% CI: 2.0–48.8) remained significantly associated with HSIL/SCC, whereas HPV52 showed a positive but non-significant association (OR = 3.8, 95% CI: 0.8–18.0). Microecological abnormality was not independently associated with HSIL/SCC after genotype adjustment (OR = 1.4, 95% CI: 0.70–2.61).

When examining predictors of HR-HPV infection (Supplementary Table S2), none of the specific microecological parameters (BV, TV, VVC) or cleanliness grades showed significant independent associations after adjustment. In genotype-specific analyses (Supplementary Tables S3–S5), HPV58 positivity was significantly associated with poorer vaginal cleanliness (Grade III: OR = 1.4, 95% CI: 1.06–1.81; Grade IV: OR = 1.7, 95% CI: 1.18–2.58), while HPV16 and HPV52 showed no significant associations with any microecological parameters. These findings suggest that the association between vaginal dysbiosis and HPV may be genotype-selective, particularly for HPV58.

## 4 Discussion

Our discussion focuses primarily on the most consistent and *a priori* hypotheses—namely, the associations of HPV16, HPV52, and HPV58 with cervical lesions—which show consistent trends across multiple analyses and are supported by biological plausibility. Secondary findings, particularly those involving less common genotypes or small subgroup analyses, are interpreted with caution and presented as exploratory observations requiring further investigation.

In a retrospective six-year study performed in Chongqing, Southwest China in 24,050 women, the overall HPV prevalence was 21.7%. Notably, it rose significantly from 7.6% in 2019 to 30.1% in 2022, stabilizing at around 25.5% in 2023–2024. The top genotypes were HPV52, HPV16, and HPV53 where HPV52 had a higher prevalence in single (19.9%) and multiple (11.2%) infections. HPV prevalence exhibited two peaks at younger and older age groups. The stepwise increase of HPV16 positivity from LSIL to SCC and the predominance of HPV52 in precancerous lesions was confirmed. Of note, it is the first large-scale study in Chongqing to systematically analyze the link between HPV genotype profiles and vaginal microecological disturbance. The pooled prevalence at 21.7%, is consistent with the multicenter average of 21.07% for urban Chinese women, suggesting the sample is representative (17, 18). The significant increase in HPV positivity between 2019 and 2022 coincided with the implementation of organized cervical cancer screening in Chongqing. One possible explanation is that the screening program uncovered a backlog of prevalent infections that had not been previously detected. However, this period also overlapped with the COVID-19 pandemic, which may have altered healthcare-seeking behavior and the demographic composition of women attending our hospital. Additionally, changes in testing volume and referral patterns may have contributed. Without individual-level data on screening history and healthcare utilization, these explanations remain speculative, and multiple factors likely contributed to the observed trend. The stabilization observed in 2023–2024 may reflect several possible factors: completion of the initial screening backlog, a return to pre-pandemic screening patterns, or the early impact of HPV vaccination in younger cohorts. However, we lack empirical data to distinguish these explanations, and they should be considered hypotheses requiring confirmation in future studies. Similar time dynamics were observed in other Chinese cities’ screening expansion (4).

A study done in Chongqing recently exhibited the relationship of vaginal dysbiosis with overall HR- HPV positivity (16). We extend these observations in three important ways: (i) genotype specific estimates for HPV52 and HPV16; (ii) a significant and strong dose response relationship with decreasing vaginal cleanliness grade (trend *p* < 0.001); and (iii) HPV16 is disproportionately enriched in severe dysbiosis (Grade IV, 13.3%) while HPV52 has broader associations with BV, TV, and VVC. If confirmed prospectively, there are clinical implications: various HPV genotypes may necessitate varied intensities of microecological intervention. Significantly, several multicenter studies previously reported HPV prevalence in other areas of China. However, none of these have captured the post-2022 stabilization period or genotype-specific HPV52/16 positivity with clinically diagnosed BV/TV/VVC in Chongqing.

A growing body of evidence indicates that vaginal dysbiosis promotes cervical carcinogenesis not merely through local inflammation, but via active modulation of host cellular signaling pathways. The shift from *Lactobacillus*-dominant communities (Community State Types I-III) to high-diversity CST IV, characterized by depletion of lactobacilli and overgrowth of anaerobes such as *Gardnerella vaginalis* and *Atopobium vaginae*, has been linked to increased susceptibility of HR-HPV persistence and high-grade cervical lesions (19, 20). Dysbiosis-associated microbial metabolites and chronic inflammatory stimuli may activate the PI3K/AKT/mTOR signaling axis—a central pathway in cervical cancer progression that promotes cell proliferation, inhibits apoptosis, and facilitates epithelial-mesenchymal transition (21). Importantly, the PI3K/AKT pathway can be modulated by long non-coding RNAs (lncRNAs) in cervical cancer cells, with dysregulated lncRNAs influencing tumor cell proliferation, invasion, and chemoresistance through miRNA sponging and epigenetic regulation (22–24). Recent studies on intestinal mucosal healing have also demonstrated that circular RNAs (circRNAs) can mediate epithelial repair through microRNA and PI3K/AKT signaling pathways (25). While these mechanistic studies were conducted in cancer cell lines and not directly in vaginal microbiome contexts, they establish a biologically plausible framework: microbial dysbiosis may create a pro-inflammatory, immunocompromised microenvironment that potentiates HPV oncoprotein activity and facilitates aberrant activation of PI3K/AKT and other oncogenic cascades (26, 27). Furthermore, recent two-sample Mendelian randomization studies have provided causal evidence linking gut microbiota composition to cancer probability through immune modulation (28, 29), supporting the broader paradigm that microbial-immune-signaling crosstalk is a conserved mechanism across mucosal sites. Extrapolating this paradigm to the cervicovaginal tract, our observation that HPV16 - the most oncogenic genotype-is disproportionately associated with the most severe dysbiosis (Grade IV) suggests a synergistic model: severe microbial disturbance may create a permissive niche for HPV16-driven carcinogenesis, while HPV52, though less intrinsically oncogenic, may exploit broader, milder dysbiotic states to establish persistent infection. This hypothesis warrants prospective testing with high-resolution microbiome sequencing and longitudinal follow-up.

The high prevalence of HPV52 in our findings is consistent with studies conducted in other cities of southern and southwestern China, while HPV16 and HPV58 predominate in northern China, suggesting a notable north–south gradient in HPV epidemiology (30). The most dominant genotype HPV52 in our research study was consistent with the study conducted in China (2015–2025) which showed that HPV52 (3.20%) was the most prevalent genotype, and other genotypes being HPV16 (2.19%) and HPV53 (1.67%) (31). This affirms the external validity of the single-center findings. Around 70% of cervical cancers are HPV16/HPV18 (12). Nevertheless, only 5.4% of our cohort was HPV18 positive. Current bivalent and quadrivalent vaccines do not prevent HPV52 or HPV53.

A number of factors are at play for the high HPV prevalence (40.3%) in women aged ≤20 years. Individuals in this age group have initiated sexual activity recently. The cervical epithelium of this age group is not as mature. Also, the local immunity against HPV infection is not that strong. Having sexual intercourse before the age of 16 was associated with HPV positivity by 2.3 times (32). The coverage of HPV vaccination in Chongqing is relatively low among young women, that is lower than 15% which is insufficient to generate herd immunity (33). The increased prevalence of low-risk HPV6 and HPV11 in this group are consistent with the peak incidence of condyloma acuminata, giving further support to vaccination with quadrivalent or 9-valent (34, 35). According to official reports, in 2022, Chongqing launched a free HPV vaccination program for junior high school girls (grade 8). By March 2023, the first-dose completion rate among eligible girls reached nearly 60%, and by April 2023, cumulative vaccination coverage among grade 8 girls were reported at 71.8%. However, coverage among older women and the general adult female population remains substantially lower. The results affirm the need for comprehensive sexual health education and timely vaccination of adolescents and young people in particular.

Women aged over 60 years (39.2%) contributed to the second peak in prevalence due to immunosenescence, which is defined as an age-related decline in immune surveillance and which impairs resistance to new HPV infection as well as clearance of latent infection (36). Women aged 61 years and above exhibited the highest values of TCT abnormalities (35.0%) among all age groups while also accounting for 43.5% of squamous cell carcinoma (SCC) cases. Thus, older women may be at extremely high-prevalence for cervical cancer but do not get screened routinely. The confirmation of similar age–genotype associations from Hubei and Sichuan recommends the implementation of age-stratified, genotype-targeted preventive strategies, including better access for screening for older adult women (5). Based on these findings, we contend that the current recommendation of 64 years as the upper age limit for cervical cancer screening warrants urgent reassessment. For older women, we advocate for individualized follow-up management using biomarker-based risk stratification strategies, rather than a one-size-fits-all approach of simply terminating screening at a fixed age (37).

Our findings provided strong evidence for the central role of HPV16 in cervical carcinogenesis in Chongqing. There was a gradual increase in the positivity rate of HPV16 with the severity of lesions. The positivity rate was 18.3% in LSIL, 36.1% in HSIL, and 60.9% in SCC. This gradient is consistent with global meta-analytic data, and confirms that HPV16 is the main driver of neoplastic progression in this area (14). While meta-analyses provide pooled estimates with potential heterogeneity, our single-center large-sample study benefits from standardized protocols and consistent quality control, which reduces heterogeneity and enhances internal validity for the Chongqing population. This high-resolution, locally consistent snapshot complements the global picture by providing precise, locally applicable data. Thus, secondary prevention efforts should place greater emphasis on HPV16 positive management. Our study shows that apart from HPV16, HPV52, 58, 53, and 33 were significantly associated with cervical lesions in Chongqing. It is noteworthy that HPV52 demonstrated a greater positivity rate than HPV16 in LSIL (26.6%) and ASCUS (26.0%). These findings are consistent with studies from Sichuan, which demonstrated a positive correlation between HPV52/58 viral loads and high-grade lesion severity (35). In SCC, HPV52 accounted for 13.0% and HPV58 for 8.7%, making them the most important HR-HPV types after HPV16 in this population. Therefore, screening programs must not limit monitoring to HPV16/18; enhanced follow-up and colposcopy for HPV52/58 positive cases are essential to prevent missed diagnoses.

A recently published multicenter study from Chongqing (*n* = 229,770) reported a similar HPV prevalence (21.41%) and a bimodal age distribution, validating the representativeness of our single-center sample (38). Notably, our study observed a comparable predominance of HPV52, but with higher resolution on lesion specific genotype distribution (ASCUS, LSIL, HSIL, SCC separately, as opposed to pooled cervical cancer cases). HPV16, not HPV52, was most strongly linked to high-grade lesions and SCC, underscoring that prevalence does not equate to oncogenic potential. The high prevalence of HPV52 in LSIL (26.6%) suggests a possible contributory role in this region, though its per-genotype oncogenic potential is lower than that of HPV16.

A healthy vaginal microbiota maintains acidic pH and enhances mucosal resistance to HPV. Dysbiosis reduces Lactobacillus, promotes inflammation, and facilitates HPV persistence (39, 40). These findings suggest a synergistic cycle in which HPV infection may be associated with dysbiosis, and dysbiosis in turn may be related to HPV persistence, collectively contributing to the development of cervical cancer. This supports integrated prevention strategies that address both HPV and the vaginal microbiota. Co-infection with other STIs may influence HPV pathogenesis by altering local immunity and promoting inflammation (41), and the higher prevalence of HPV52 in women with BV, TV, and VVC in our cohort may reflect such interactions, though our cross-sectional design limits causal interpretation.

## 5 Limitations

Furthermore, limitations exist regarding the interpretation of the study results. (1) We did not have data on participants’ HPV vaccination status. In the post-2022 period, vaccination coverage may have increased, potentially altering genotype distribution in younger women. Without this data, attributing changes in prevalence to screening alone is speculative. (2) TCT was performed on only 50.1% of HPV-positive women, which may introduce selection bias, as testing decisions were influenced by clinical presentation and departmental protocols. This limits the representativeness of our cytology findings. (3) We lacked information on sexual behavior, contraceptive use, smoking, or parity—all of which are known confounders for HPV acquisition and persistence. The absence of these variables may have introduced residual confounding in our analyses. (4) Our vaginal microecology assessment (wet mount microscopy and dry chemistry) provides a clinically oriented phenotypic snapshot but does not characterize the full microbial community composition via modern metagenomic methods such as 16S rRNA sequencing. Higher-resolution microbial profiling in future studies could reveal genotype-specific microbial signatures that our current methods cannot detect. (5) This single center urban study may not represent rural or other southwestern populations. However, running the study consecutively over six-years meant we could maintain consistent quality control, which improves internal consistency for this population. Our hospital acts as both a community medical facility and atertiary hospital for abnormal cervical screening cases, which may elevate the observed HPV prevalence relative to routine community screening. The steady HPV detection rate between 2023 and 2024 merely reflects saturated screening among our hospital’s covered population instead of stabilized infection levels across the whole community. Furthermore, these urban single-center findings cannot be extended to rural groups in Southwest China or other provinces, given notable regional disparities in medical access, screening coverage and HPV genotype distribution. (6) Only 23 SCC cases were identified. Statistical power was limited for reliable genotype-specific estimates in advanced lesions. This is reflected in wide confidence intervals. (7) The cross-sectional design precludes assessment of HPV persistence or clearance, critical for distinguishing transient infections. Consequently, we cannot determine temporal sequence or adjust for unmeasured confounders. Longitudinal studies are required to address these limitations. (8) In this exploratory epidemiological study, a large number of pairwise comparisons were performed across HPV genotypes, age groups, and cytological categories without correction for multiple testing. This increases the probability of occurrence of type I error I, meaning that some statistically significant findings may have arisen by chance. Therefore, secondary genotype–disease associations should be interpreted cautiously and warrant further validation in independent cohorts.

The fact that only 50.1% of HPV-positive women underwent TCT represents a significant limitation. TCT was not performed uniformly, which may introduce selection bias. Women who underwent TCT may have differed systematically from those who did not—for example, they may have been more symptomatic, had higher-risk genotypes, or had greater healthcare access. Therefore, our estimates of genotype-specific associations with cervical lesions may not be representative of the entire HPV-positive population and should be interpreted with caution.

Although multivariable logistic regression adjusting for age and HPV genotype attenuated the association between vaginal microecological abnormality and HSIL/SCC (adjusted OR = 1.35, 95% CI: 0.70–2.61), residual confounding from unmeasured factors (e.g., sexual behavior, smoking, vaccination status) cannot be excluded. Our findings are consistent with recent studies suggesting that the effect of vaginal microbiota on cervical lesion progression may be largely mediated by HPV genotype rather than being an independent driver (39, 40).

## 6 Conclusion

This six-year study of 24,050 women in Chongqing reveals that HPV52 predominates in both infections and precancerous lesions, diverging from the global HPV16/18 paradigm. We provide the first evidence in Chongqing that the association between vaginal dysbiosis and HR-HPV is genotype specific and dose-dependent. HPV16 is enriched in severe dysbiosis, while HPV52 shows broader associations across BV, TV, and VVC. These findings support regionally tailored strategies, including prioritizing the 9-valent vaccine, prioritizing colposcopy over cytology-based screening alone for HPV52/58-positive women. Additionally, they offer a high-resolution reference for genotype-specific HPV surveillance in Southwest China and generate testable hypotheses for future multicenter studies.

## Data Availability

The original contributions presented in the study are included in the article/Supplementary material, further inquiries can be directed to the corresponding authors.
