# Supplementary material for: HPV52 predominates in cervical infections and precancerous lesions in Chongqing, China: a 6-year study linking genotypes to vaginal microecology
Source: Front Public Health. 2026 Aug 14;14:1879232. doi: 10.3389/fpubh.2026.1879232 (PMC13522131; doi:10.3389/fpubh.2026.1879232)
Supplement: Supplementary file 1 [file Supplementary_file_1.docx]

**Table S1.** Multivariable Logistic Regression for HSIL/SCC

| **Variable** | **Model 1 OR (95% CI)** | **Model 1 P** | **Model 2 OR (95% CI)** | **Model 2 P** | **Model 3 OR (95% CI)** | **Model 3 P** |
| --- | --- | --- | --- | --- | --- | --- |
| **Microecological abnormality** | 1.516 (0.795–2.891) | 0.2069 | 1.360 (0.706–2.622) | 0.3582 | 1.353 (0.701–2.611) | 0.3675 |
| Age (per year) | 1.056 (1.037–1.076) | <0.001 | 1.057 (1.037–1.077) | <0.001 | 1.058 (1.038–1.079) | <0.001 |
| Calendar year (per year) | 0.738 (0.621–0.877) | <0.001 | 0.738 (0.619–0.881) | <0.001 | 0.744 (0.623–0.887) | 0.0010 |
| HPV16 (ref: low risk) | — | — | 13.144 (2.978–58.016) | <0.001 | 15.286 (3.413–68.458) | <0.001 |
| HPV52 (ref: low risk) | — | — | 3.370 (0.721–15.752) | 0.1225 | 3.829 (0.813–18.040) | 0.0896 |
| HPV58 (ref: low risk) | — | — | 8.845 (1.817–43.067) | 0.0070 | 9.943 (2.027–48.772) | 0.0046 |
| Other HR-HPV (ref: low risk) | — | — | 2.491 (0.559–11.102) | 0.2312 | 2.762 (0.616–12.375) | 0.1842 |
| Double infection (ref: Single infection) | — | — | — | — | 0.693 (0.339–1.415) | 0.3135 |
| Triple+ infection (ref: Single infection) | — | — | — | — | 0.596 (0.237–1.504) | 0.2733 |

Population: HPV-positive women with TCT results (N = 1835); Outcome: HSIL/SCC vs NILM/ASCUS/LSIL; Microecological abnormality: BV/TV/VVC positive or vaginal cleanliness grade ≥ III

**Table S2.** Logistic Regression for HR-HPV Infection

| **Variable** | **Model 1 OR (95% CI)** | **Model 1 P** | **Model 2 OR (95% CI)** | **Model 2 P** | **Model 3 OR (95% CI)** | **Model 3 P** |
| --- | --- | --- | --- | --- | --- | --- |
| **BV infection (vs NC)** | 1.072 (0.690–1.665) | 0.7585 | — | — | 1.110 (0.711–1.734) | 0.6465 |
| **TV infection (vs NC)** | 1.021 (0.442–2.357) | 0.9613 | — | — | 1.085 (0.467–2.520) | 0.8495 |
| **VVC infection (vs NC)** | 0.715 (0.520–0.984) | 0.0395 | — | — | 0.745 (0.537–1.035) | 0.0791 |
| Cleanliness Grade III (vs I/II) | — | — | 0.928 (0.771–1.116) | 0.4253 | 0.949 (0.785–1.148) | 0.5917 |
| Cleanliness Grade IV (vs I/II) | — | — | 0.803 (0.605–1.065) | 0.1278 | 0.831 (0.621–1.112) | 0.2134 |
| Age (per year) | 1.001 (0.994–1.008) | 0.7854 | 1.002 (0.995–1.009) | 0.5463 | 1.001 (0.994–1.008) | 0.7342 |
| Calendar year (per year) | 1.038 (0.974–1.106) | 0.2469 | 1.041 (0.976–1.110) | 0.2205 | 1.042 (0.977–1.111) | 0.2075 |

Population: HPV-positive women with microecology data (N = 3183); Outcome: HR-HPV positive (1) vs LR-HPV only (0)

**Table S3.** Logistic Regression for HPV16 vs Others

| **Variable** | **Model 1 OR (95% CI)** | **Model 1 P** | **Model 2 OR (95% CI)** | **Model 2 P** | **Model 3 OR (95% CI)** | **Model 3 P** |
| --- | --- | --- | --- | --- | --- | --- |
| **BV infection (vs NC)** | 0.824 (0.467–1.455) | 0.5051 | — | — | 0.767 (0.432–1.361) | 0.3643 |
| **TV infection (vs NC)** | 1.801 (0.777–4.171) | 0.1699 | — | — | 1.640 (0.702–3.830) | 0.2528 |
| **VVC infection (vs NC)** | 0.928 (0.601–1.433) | 0.7353 | — | — | 0.854 (0.548–1.332) | 0.4872 |
| Cleanliness Grade III (vs I/II) | — | — | 1.183 (0.940–1.490) | 0.1520 | 1.206 (0.953–1.527) | 0.1188 |
| Cleanliness Grade IV (vs I/II) | — | — | 1.264 (0.887–1.802) | 0.1950 | 1.286 (0.892–1.852) | 0.1773 |
| Age (per year) | 0.997 (0.988–1.006) | 0.4874 | 0.996 (0.988–1.005) | 0.4193 | 0.996 (0.988–1.005) | 0.4071 |
| Calendar year (per year) | 0.907 (0.840–0.980) | 0.0132 | 0.907 (0.839–0.980) | 0.0136 | 0.906 (0.839–0.980) | 0.0132 |

Population: HPV-positive women with microecology data (N = 3183); Outcome: HPV16 positive = 1, all other HPV types = 0 (n = 398)

**Table S4.** Logistic Regression for HPV52 vs Others

| **Variable** | **Model 1 OR (95% CI)** | **Model 1 P** | **Model 2 OR (95% CI)** | **Model 2 P** | **Model 3 OR (95% CI)** | **Model 3 P** |
| --- | --- | --- | --- | --- | --- | --- |
| **BV infection (vs NC)** | 1.139 (0.756–1.716) | 0.5345 | — | — | 1.126 (0.743–1.705) | 0.5771 |
| **TV infection (vs NC)** | 1.083 (0.488–2.405) | 0.8444 | — | — | 1.076 (0.482–2.404) | 0.8574 |
| **VVC infection (vs NC)** | 1.274 (0.925–1.755) | 0.1384 | — | — | 1.258 (0.906–1.749) | 0.1710 |
| Cleanliness Grade III (vs I/II) | — | — | 1.082 (0.904–1.295) | 0.3904 | 1.051 (0.873–1.264) | 0.6012 |
| Cleanliness Grade IV (vs I/II) | — | — | 1.047 (0.789–1.390) | 0.7503 | 1.000 (0.747–1.339) | 0.9998 |
| Age (per year) | 1.001 (0.994–1.008) | 0.7864 | 1.000 (0.994–1.007) | 0.9070 | 1.001 (0.994–1.008) | 0.8100 |
| Calendar year (per year) | 1.028 (0.965–1.095) | 0.3957 | 1.029 (0.965–1.096) | 0.3821 | 1.029 (0.966–1.097) | 0.3718 |

Population: HPV-positive women with microecology data (N = 3183); Outcome: HPV52 positive = 1, all other HPV types = 0 (n = 718)

**Table S5.** Logistic Regression for HPV58 vs Others

| **Variable** | **Model 1 OR (95% CI)** | **Model 1 P** | **Model 2 OR (95% CI)** | **Model 2 P** | **Model 3 OR (95% CI)** | **Model 3 P** |
| --- | --- | --- | --- | --- | --- | --- |
| **BV infection (vs NC)** | 0.885 (0.481–1.627) | 0.6931 | — | — | 0.771 (0.417–1.426) | 0.4076 |
| **TV infection (vs NC)** | 1.536 (0.589–4.001) | 0.3800 | — | — | 1.266 (0.482–3.329) | 0.6322 |
| **VVC infection (vs NC)** | 0.410 (0.208–0.811) | 0.0103 | — | — | 0.350 (0.176–0.697) | 0.0028 |
| Cleanliness Grade III (vs I/II) | — | — | 1.285 (0.987–1.672) | 0.0623 | 1.386 (1.061–1.811) | 0.0167 |
| Cleanliness Grade IV (vs I/II) | — | — | 1.558 (1.063–2.283) | 0.0231 | 1.743 (1.178–2.580) | 0.0054 |
| Age (per year) | 1.004 (0.994–1.013) | 0.4486 | 1.004 (0.995–1.013) | 0.3867 | 1.002 (0.993–1.012) | 0.6062 |
| Calendar year (per year) | 0.967 (0.886–1.055) | 0.4492 | 0.962 (0.881–1.051) | 0.3926 | 0.962 (0.881–1.051) | 0.3918 |

Population: HPV-positive women with microecology data (N = 3183); Outcome: HPV58 positive = 1, all other HPV types = 0 (n = 306)
